# Natural compounds for diabetes-related wounds or ulcers therapy: Evidence from preclinical and clinical studies

**DOI:** 10.1016/j.jgr.2026.100990

**Published:** 2026-02-10

**Authors:** Jia Teng, Guanchi Yan, Ming Yang, Shuangyue Wang, Yuezhu Zhao, Yanyan Wang, Jia Mi

**Affiliations:** a College of Traditional Chinese Medicine, Changchun University of Chinese Medicine, Changchun, China; b The Affiliated Hospital to Changchun University of Chinese Medicine, Changchun University of Chinese Medicine, Changchun, 130021, China

**Keywords:** Traditional medicines, Natural compounds, Clinical studies, Preclinical studies, Diabetes-related wounds or ulcers

## Abstract

**Background:**

Diabetes-related wounds or ulcers are one of the complications of diabetes that can cause a variety of functional losses. Treatment of diabetic skin ulcer (DSU) focuses on alleviating foot pressure and enhancing local circulation. Despite the availability of these treatments, the number of drugs for wound application is limited. In recent years, natural compounds obtained from plants have demonstrated positive effects on DSU and have effective in preclinical and clinical studies.

**Purpose:**

The objective of the study was to assess the diabetes-related wounds or ulcers of natural compounds and the potential mechanisms.

**Study design:**

and Methods: The study searched PubMed database up to January 2025.

**Results:**

In the review, the efficacy and safety of natural compounds in treating diabetes-related wounds or ulcers are summarized from 42 preclinical and 13 clinical studies.

**Conclusion:**

Overall, these preclinical and clinical studies advocate for the use of natural compounds in diabetes-related wounds or ulcers, indicating that ursolic acid, paeoniflorin, and *Curcuma zedoaria* polysaccharide (ZWP) could be effective and well-tolerated potential medications.

## Graphical abstract

**Figure:**
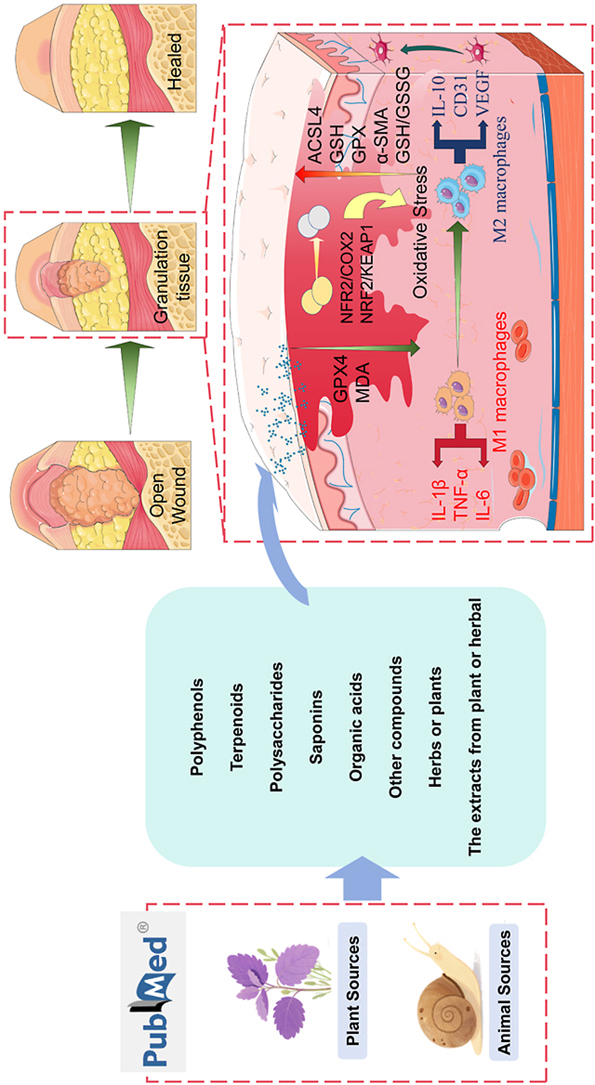

## 1 Introduction

In 2024, the number of adults aged 20-79 with diabetes worldwide reached 589 million, it is estimated that by 2050, the total number of diabetes patients will climb to 853 million [1]. Diabetes-related wounds or ulcers are the primary contributors to disability, mortality, and healthcare burden in diabetes. Diabetic skin ulcer (DSU) impact about 20 million people worldwide annually, which can result in infections, hospitalizations, amputations, and death. Following treatment, the recurrence rate of diabetic foot ulcers is 65% within three to five years, with 20% rate of lower limb amputation and five years mortality rate of 50% to 70% [2].

In recent decades, wound healing and limb preservation in diabetes have garnered much attention because of their potentially implications. The management of diabetes-related wounds or ulcers faces challenges include persistent inflammation, inadequate blood vessel formation, and hindered skin cell regeneration. The goal of doctors to promote the healing of diabetes-related wounds or ulcers by reducing the time needed for wound closure, improving the cost-effectiveness of treatments, and increasing limb preservation. In the past few years, it has been recognized by researchers that a combination of methods, including treatment of diabetes, surgical debridement, anti-infection, hyperbaric oxygen therapy, decompression, and topical dressings. In recent, Recombinant human platelet-derived growth factor-BB (Becaplermin, Regranex®) has been approved by the FDA for the treatment of diabetic foot ulcers [3]. These interventions are complex and costly, and they do not influence the recurrence of the disease. The exploration of new treatments for diabetes-related wounds or ulcers is beneficial by the limitations of surgical debridement and limited options for drug therapy.

Ethnomedical systems worldwide have long utilized specific plants to manage diabetes and its complications, including chronic wounds. Plants like *Curcuma zedoaria*, traditionally valued for its anti-inflammatory and wound-healing properties, is rich in bioactive polysaccharides. *Paeonia lactiflora*, documented in traditional Chinese medicine for promoting blood circulation and tissue repair, contains key terpenoids such as paeoniflorin. *Glycyrrhiza glabra*, recognized across cultures for its soothing and anti-infective actions on skin ailments, is a primary source of saponins like glycyrrhizic acid and glycyrrhetinic acid. Other plants mentioned, including *Teucrium polium*, *Gynura divaricata*, and *Moringa oleifera*, also have established histories in local healing practices for ulcers and skin injuries. Modern preclinical and clinical studies now validate these traditional applications by identifying the specific compounds within these plants-such as polyphenols, terpenoids, polysaccharides, and saponins-that drive their efficacy against diabetes-related wounds. This convergence of traditional knowledge and scientific evidence highlights the therapeutic potential of these botanical compounds.

Natural compounds can be utilized in diabetes-related wounds or ulcers. These compounds have various biological effects, including antioxidant, anti-inflammatory, anti-bacterial, promotion of angiogenesis, regulation of gut flora, and immunity enhancement. Specifically, their multifaceted actions include promoting wound closure, promoting collagen synthesis, reducing inflammation, and promoting blood vessel formation. The study intends to review the effects of natural compounds in diabetes-related wounds or ulcers and describe the potential pharmacological mechanisms.

## 2 Methods

### 2.1 Search strategy

The study searched PubMed database until January 2025. The keywords included natural compounds, diabetes-related wounds or ulcers, plants, and herbs. The search strategy in Supplementary Table 1.

### 2.2 Inclusion and exclusion criteria

The study included preclinical or clinical studies of natural compounds for the treatment of diabetic wounds or ulcers, with full text available. The source and dose of natural compounds were recorded in the paper. The exclusion criteria for the study included: (1). the preparations of herbs or compounds; (2). the full text was unavailable; (3). the data was incomplete, unavailable, or incorrect; (4). the same data published by the same author in different journals; (5). the unclear ingredients.

## 3 Results

**Fig. 1:**
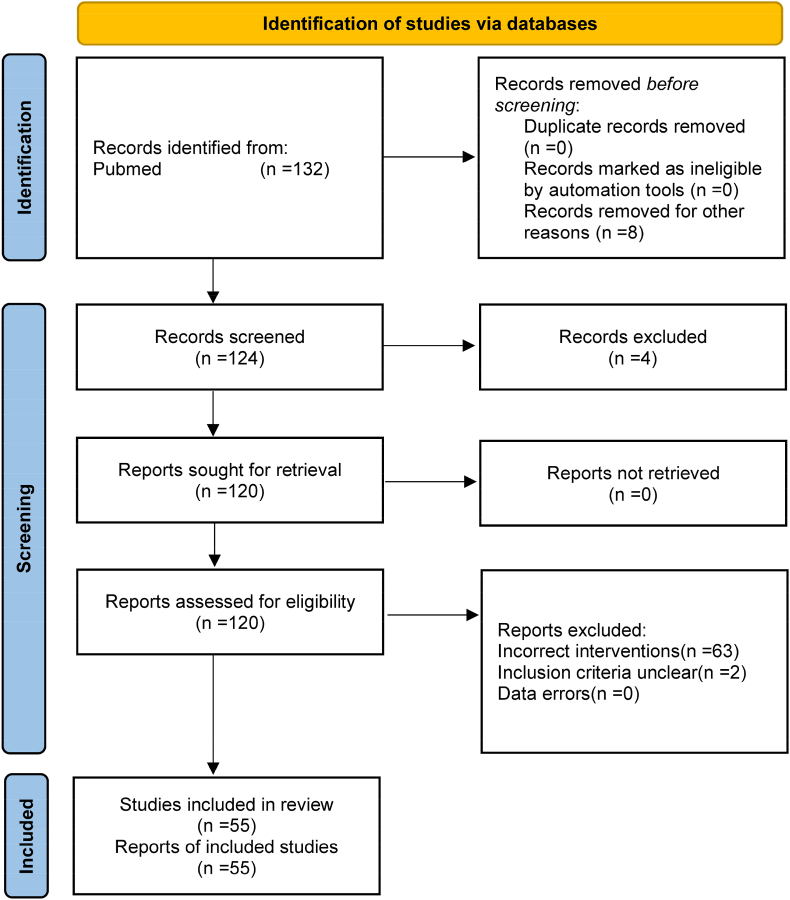
Study selection method and analysis flow chart.

The study retrieved a total of 132 papers. According to the inclusion and exclusion criteria, 77 papers were finally excluded and 55 papers were included. There were 42 papers of preclinical studies [[4], [5], [6], [7], [8], [9], [10], [11], [12], [13], [14], [15], [16], [17], [18], [19], [20], [21], [22], [23], [24], [25], [26], [27], [28], [29], [30], [31], [32], [33], [34], [35], [36], [37], [38], [39], [40], [41], [42], [43], [44], [45]] and 13 papers of clinical studies [[46], [47], [48], [49], [50], [51], [52], [53], [54], [55], [56], [57], [58]]. The research selection process was shown in Fig. 1. The results of preclinical studies were shown in Table 1. The results of clinical studies were shown in Table 2.

**Table 1:** Reports preclinical studies of natural compounds for the treatment of diabetes-related wounds or ulcers.

Name of compound	Molecular structure diagram	Modeling method	Wound structure	Mouse model	Methods of drug administration in the experimental group	Results	PubChem Compound CID
ZWP [38]	NA	Intraperitoneal injection of STZ (60 mg/kg) dissolved in 50 mM sodium citrate buffer (pH 4.5)	The upper dorsal skin was excised using a circular seal (18 mm diameter) to create a standardized full-thickness wound	Female SD rats	The wounds were covered with a composite sponge containing 100 μg of ZWP and PRP-Exos/ZWP, respectively.	1. Treatment with ZWP alone or the PRP-Exos/ZWP combination was found to effectively decrease blood glucose levels in diabetic mice, while no significant impact on body weight was observed. 2. Treatment with ZWP or the combination of PRP-Exos and ZWP can enhance the rate of wound contraction, increase epidermal thickness, and promote collagen synthesis and deposition.	NA
EGCG [19]		Intraperitoneal injection of STZ(100 mg/kg)	Two circular 8-mm full-thickness wounds were created on the back of each mouse using a dermal biopsy punch	Male ICR mice	DM mice were treated with 10 mg/mL EGCG	1. EGCG can decrease the levels of inflammatory factors, including IL-1β, TNF-α, and IL-6, as well as inhibit the accumulation of macrophages in the wound tissue of diabetic mice. 2. EGCG promotes wound healing by suppressing the Notch signaling pathway.	65064
IGLR [15]	NA	NA	Sterile needle biopsy tools were used to create 8-mm wounds on the back of mice	Male db/db mice	After dilution in 0.3% CMC-Na, the mice were given low (0.1 g/kg/d) and high (0.4 g/kg/d) doses by gavage	IGLR inhibits oxidative stress and inflammation by modulating the NRF2/COX2 signaling pathway. Additionally, IGLR suppresses oxidative stress through the NRF2/KEAP1 signaling pathway and exerts its effects via a paracrine mechanism to activate fibroblasts.	NA
*Teucrium polium*(*T. polium)* [13]	NA	Alloxan (120 mg/kg) dissolved in normal saline was injected subcutaneously	Two full-thickness incisions (2 × 2 cm in length) were made on the back of the chest with a sterile surgical scalpel	Male Wistar rats	The wound was treated locally with 10%*T. polium* extract ointment	10% *T. polium* extract ointment can improve the wound closure rate.	NA
HLG [22]	NA	STZ(40 mg/kg/day) was injected intraperitoneally for 6 days	A sterile biopsy punch was utilized to create a 6 mm diameter wound	Male C57BL/6J mice	The extract preparation was applied to the wound	HLG can improve the wound healing rate.	NA
AFG [45]	NA	STZ-induced type Ⅰ diabetic SD rats or db/db mice	A full-thickness circular skin wound measuring 6 mm in diameter was created on the dorsal side using a disposable biopsy punch	SD rats	The dual network hydrogel biomaterials consisting of AFG and GelMA were applied externally to the wounds	AFG/GelMA can improve the wound healing effect and increase the wound closure rate.	NA
CGE [12]	NA	NA	A 1 cm^2^ wound was created	Male db/db mice	CGE was applied to the wound	1. CGE can increase wound healing rate, collagen fiber density and anti-inflammatory gene level. 2. CGE could up-regulate M2 macrophages.	NA
PE [32]	NA	Intraperitoneal injection of STZ (50 mg/kg)	Two incisions 1 cm long and 0.3 cm deep were made with a scalpel	Male ICR mice	100 μg/g PE gel and 150 μg/g PE gel were applied topically to the incision	PE can improve the wound healing rate and collagen infiltration score.	NA
Orientin [40]		Intraperitoneal injection of STZ (100 mg/kg)	Two full-thickness skin incisions were made on both sides of the dorsal trunk, with an area of 0.385 cm^2^ and a diameter of 0.7 cm	NA	The once-daily intraperitoneal injection dose was 10 mg/kg Orientin solution	1. Orientin increased the expression of ferroptosis-related protein ACSL4 and decreased the expression of ferroptosis-related protein GPX4. 2. Orientin can effectively reduce the accumulation of mitochondrial reactive oxygen species. 3. Orientin can promote angiogenesis and wound healing.	5281675
MOL [10]	NA	Intraperitoneal injection of STZ (35 mg/kg b.w.) dissolved in 0.1 M citrate buffer (pH 4.5)	A biopsy punch with a diameter of 6 mm was used to create wounds on both sides of the back. A 1 × 1 cm^2^ abrasion wound was created on the back using coarse sandpaper and acetone	SD rats	MOL membranes of 0.1%, 0.5%, and 1% were applied externally, respectively	1. Topical application of 1.0.5% MOL membrane can improve the wound healing rate. 2. MOL membrane can make collagen deposition more concentrated and fibers more organized in the wound. MOL extract could up-regulate Col1a1 And the expression of IL-6 and MCP-1.	NA
HA-PF [39]		Intraperitoneal injection of STZ (55 mg/kg)	Two full-thickness wounds of about 0.3 cm^2^ were created deep in the dermis using sterile surgical scissors	Male C57BL/6J mice	External application of HA-PF (100 μL)	HA-PF can improve wound healing. 2.HA-PF can reduce inflammatory infiltration, improve epidermal hyperplasia, and promote the growth of new blood vessels. HA-PF could improve the distribution of collagen. 4.HA-PF could increase the number of M2 phenotype and enhance the expression of CD31 and VEGF.	442534
HST [41]		Intraperitoneal injection of STZ (30 mg/kg)	Two full-thickness round wounds with a diameter of 2 cm were created in the dorsal region of the mice	Male SD rats	100 μL of gel containing 10 μM HST was applied to the wound	1. The GSH/GSS ratio increased significantly after HST treatment, indicating a dose-dependent therapeutic effect. In addition, HST also effectively inhibited the increase of MDA level after erastin stimulation. 2.HST treatment significantly restored cell viability and morphology. 2. HST treatment could counteract the erastin-induced inhibition of angiogenesis. 4.HST has the potential to promote wound healing by inhibiting iron ptosis and restoring mitochondrial function.	72281
*Rosmarinus officinalis L.* extract [4]	NA	Intraperitoneal injection of Alloxan (150 mg/kg)	A full-thickness skin excision wound with a diameter of 15 mm was created on the back of the mouse in a symmetrical position on both sides of the spine	Male BALB/c mice	1. 0.2 mL of 10% (v/v) aqueous extract was intraperitoneally injected once a day. 2. Apply 25 μL of pure essential oil to the incision, twice a day.	The extract could down-regulate the blood glucose and body weight of diabetic mice, and increase the weight of granulation tissue and the percentage of wound contraction.	NA
Ast [7]	NA	Intraperitoneal injection of STZ (65 mg/kg) dissolved in 50 mM citrate buffer (pH 4.5-4.8)	A needle biopsy device with a diameter of 8 mm was used to create the wound	Male BALB/c mice	The wound was covered with core-shell SF@chitosan/ZnO/Ast	1. This fibrous membrane can improve the wound contraction rate. 2. Ast-0.5 showed the strongest inhibitory effect on *Staphylococcus aureus*, and Ast showed the strongest inhibitory effect on *Escherichia coli*. 3. Ast can promote angiogenesis.	NA
MOL [26]	NA	Induced by a combination of STZ and NAD	A sterile 6 mm biopsy punch was used to create a wound with a diameter of 6 mm and a depth of 2 mm	Male Wistar rats	Aqueous fractions of each concentration (0.5%, 1%, and 2% w/w) were dissolved in a small amount of distilled water and applied to the wound	1. MOL extract showed antibacterial activity against *Staphylococcus* aureus, *Pseudomonas aeruginosa*, and *Escherichia coli*. 2. MOL extract was able to increase the rate of contraction. 3. MOL extract could shorten the epithelialization time. 4.MOL extract could down-regulate the levels of cytokines.	NA
Cur [23]		A single high dose of STZ was administered	A 1 × 1 cm^2^ wound was created in the dorsal thoracolumbar region of the mouse	Female BALB/c mice	Apply CNPs@GMs directly to the wound	1. CNPs@GM significantly increased GSH levels and decreased ROS levels. 2. CNPs@GM can promote the migration of BJ Cells. 3. The wound size was significantly reduced, and the wound contraction rate was significantly increased. 4. CNPs@GMs/hydrogel can increase the expression levels of GPX and α-SMA. CNPs@GM made more, better and more uniform neovascularization in the wound, and the number of Ki 67 positive cells was increased.	969516
UA [24]		Mouse model of type Ⅰ diabetes from previous studies	A standardized full-thickness wound with a diameter of 10 mm was created using a circular skin sampler	NA	Different concentrations of UA (0.1%, 0.2%, 0.5% (w/v)) were added to chitosan-polyvinyl alcohol solution and applied to the wound	1. UA could reduce the expression levels of IL-6 and TNF-α. 2. UA can improve the wound closure rate, hair follicle regeneration rate, collagen content and CD31 positive tissue coverage area. 3. UA can regulate the transition of macrophage phenotype from M1 to M2.	64945
AVE [16]	NA	Intraperitoneal injection of STZ (60 mg/kg)	A circular biopsy punch with a diameter of 10 mm was used to create a full-thickness skin wound	Male SD rats	The wound was covered with a polyethylene oxide/polyurethane nanofiber membrane loaded with both nanoparticles and AVE	1. AVE impregnated nanofiber pad can increase the percentage of wound contraction. 2. AVE impregnated nanofiber pad can reduce serum glucose and inflammatory cytokines levels. 3. AVE impregnated nanofiber pad has good antibacterial properties.	NA
Egg yolk oil [21]	NA	Single injection of STZ(65 mg/kg) dissolved in 0.1 moL/l sodium citrate buffer (pH4.2)	Two circular full-thickness 6 mm diameter excision wounds were created in the dorsal chest using a sterile needle biopsy device	Female SD rats	Egg yolk oil was applied to the wound once a day for 14 days	1. Egg yolk oil can shorten the re-epithelialization time. 2. Egg yolk oil can accelerate wound contraction. 3. Egg yolk oil can increase the wound vascular density. 4. Egg yolk oil reduced mast cell number and promoted mast cell degranulation.	NA
Banyan tree, onion plant extracts [42]	NA	Intraperitoneal injection of STZ (65 mg/kg)	A full-thickness skin wound was created using a circular biopsy punch with a diameter of 10 mm	Male SD rats	Wounds were covered with sericine hydrogels loaded with plant extracts	1. The plant extract hydrogel could increase the levels of IL-10 and TIMP, and decrease the levels of TNF-α, IL-6, MMP-2 and MMP-9. 2. Plant extract hydrogel can improve wound contraction rate.	NA
HEA [5]	NA	Intraperitoneal injection of STZ (60-70 mg/kg)	A 5 mm diameter wound was created on the back	Male ICR mice	10 μl of the extract was applied daily to the wound	HAE can accelerate wound tissue repair.	NA
OAT [34]	NA	Intraperitoneal injection of NAD (110 mg/kg) followed by STZ (50 mg/kg)	An open full-thickness wound of 2 cm × 2 cm was created on the back of the rat	Male Wistar rats	The wound was covered with a KGM + KER + OAT stent, which was changed every 3 days	1. KGM + KER + OAT scaffold can inhibit the growth of *Staphylococcus aureus*. 2. KGM + KER + OAT can promote wound contraction and collagen deposition. 3. KGM + KER + OAT can increase collagen content and the number of fibroblasts in granulation tissue.	NA
TMP [11]		Intraperitoneal injection of STZ (20%, w/v) for five consecutive days (dose 55 mg/kg/day)	Two full-thickness circular wounds of approximately 8 mm in diameter were made on the surface of the mouse body	Male C57BL/6 mice	Topical application of 500 μL TMP-HA (containing 250 μmol/L TMP) to the wound	1. TMP-HA could reduce the number of M1 macrophages and increase the number of M2 macrophages. 2. TMP-HA could down-regulate STAT1 activity and iNOS expression, and up-regulate STAT6 activity and Arg-1 expression. 3. TMP-HA can up-regulate the expression of CD31, VEGF, α-SMA and COL1a1.	14296
C66[18]	NA	Intraperitoneal injection of STZ (50 mg/kg)	Circular full-thickness skin excision wounds with a diameter of 6 mm were created in the middle of each side of the spine	Male C57BL/6 mice	Low and high doses of C66 were administered by intraperitoneal injection once a day	1. C66 can improve the healing rate of diabetic wound. 2. C66 can increase the rate of re-epithelialization. 3. C66 can promote angiogenesis and increase the proportion of proliferating cells in the wound. 4. C66 can increase the level of miR-146a in wound and inhibit the mRNA expression of inflammation-related cytokines such as TNF-α, IL-6 and IL-8. 5. C66 can inhibit the activity of NF-κB by targeting IRAK1.	NA
SGAG [36]	NA	Intraperitoneal injection of STZ (50 mg/kg)	A sterile 6 mm biopsy punch was used to create the wound	Male Kunming mice	Low and high concentrations of snail glycosaminoglycan were applied directly to the wound surface for 5 consecutive days	1. The enhancement of vascular density by SGAG. 2. SGSG can promote angiogenesis. 3. SGAG can reduce edema and inflammation in the early stage of trauma.	NA
Cur [14]		Intraperitoneal injection of STZ (60 mg/kg)	Two full-thickness skin wounds with a diameter of 15 mm were made on both sides of the back of the rat	Male SD rats	Wounds were treated with PCSA hydrogel once every two days	1. PCSA hydrogel can promote the process of epithelization, promote the formation of granulation tissue, promote the formation of new blood vessels, hair follicle formation, and improve the speed of wound healing. 2. PCSA hydrogel can reduce the inflammatory response at the wound site. 3. PCSA hydrogel can convert pro-inflammatory M1 macrophages into anti-inflammatory M2 macrophages. 4. PCSA hydrogel can reduce ROS level, down-regulate IL-1β and up-regulate CD31 expression.	969516
CG [25]	NA	Intraperitoneal injection of STZ (120 mg/kg)	An open circular wound of 1 cm in diameter was created with a biopsy perforator in the interscapular region of the back	C57BL/6 mice and Wistar rats	AgNP-impregnated PEG hydrogel was applied to the wound every other day	1. CGH can scavengers free radicals. 2. CGH has an inhibitory effect on *Escherichia coli*. 3. CGH can improve ECM formation, increase collagen and fibroblast proliferation, reduce the levels of inflammation-related cytokines such as IL-6 and TNF-α, and increase the levels of anti-inflammatory cytokines such as IL-10. 4. The use of CGH increased GSH level and collagen area percentage.	NA
PvE-3[35]	NA	NA	Two full-thickness wounds were created on the back of the mouse using a 6-mm diameter skin biopsy pricker	Male db/db mice and db/m mice	Each wound was applied with 100 μL of PvE-3 solution at a concentration of 160 μg/mL and covered with a Tegaderm membrane	PvE-3 can accelerate wound healing and promote neovascularization.	NA
AVE [8]	NA	Intraperitoneal injection of STZ (40 mg/kg bw)	NA	Male Wistar rats	*A. vera* gum extract (300 mg/kg), *A. vera* carbohydrate enriched fraction (54 mg/kg), *A. vera* peptide/polypeptide fraction (0.45 mg/kg) were given orally.	AVE can reduce the levels of FPG、TG and CHOL. AVE reduced the levels of Apo-B and Apo-E.	NA
Bitter melon extract [30]	NA	Intravenous injection of STZ solution (50 mg/kg) dissolved in 10 mM citrate buffer (pH 5.0)	Two bilateral wound chambers were implanted on either side of the thoracolumbar region	Male SD rats	Local injection of bitter melon extract (0.2 mL)	1. Bitter melon extract can prevent the degeneration of granulation tissue induced by diabetes and increase the thickness of granulation tissue. 2. Bitter melon extract could increase CD68 positive cells, myofibroblasts and VEGF levels. 3. Bitter melon extract could increase the density of CD34 positive cells and blood vessels in granulation tissue.	NA
*Ginkgo biloba* Extract [9]	NA	Alloxan (130 mg/kg) intraperitoneal injection	An oval open wound measuring 2 cm × 1 cm was created using surgical scissors	Male Wistar rats	A topical cream of *Ginkgo biloba* extract (0.13 mg/mm^2^) was applied to the wound once every two days	1. *Ginkgo biloba* extract can promote the integrity of epidermal layer, promote dermal regeneration, and improve the epithelization score. 2. *Ginkgo biloba* extract improved the arrangement of collagen in regenerated skin tissue.	NA
HCE [6]	NA	Intraperitoneal injection of STZ (50 mg/kg)	Two circular wounds were created on either side of the thoracodorsal region of the rat using a sterile 6 mm biopsy punch	Adult Wistar rats	Topical 15% (w/w) HCE ointment was applied to the wound	1. HCE can increase the content of HYP. 2. HCE can reduce the level of MDA. HCE can increase the expression of MMP-1.	NA
APN [29]		Intraperitoneal injection of STZ(60 mg/kg)	A circular excision wound was made on the back of the rat	Wistar rats	Apply GGCH-HGs directly to the wound	1. GGCH-HGs can promote wound contraction and contraction. 2. GGCH-HGs can increase the weight of granuloma. 3. GGCH-HGs can promote the healing of diabetic and normal wounds	5280443
GL、GA [43]		STZ (35 mg/kg) dissolved in 0.01 M sodium citrate buffer (pH 4.4) was injected intraperitoneally	A full-thickness skin wound with a diameter of 7 mm was inoculated on the back of the rat.	SD rats	GA (100 μg/mg), GL (100 μg/mg), Cu-SD (900 μg/mg), GL@Cu-SD (200 μg/mg) and GA@Cu-SD (900 μg/mg) were used for topical application and changed once every two days	1. GL and GA loaded with Cu-SD can inhibit α-GLU activity in vitro and have antibacterial effect on *Escherichia coli* and *Staphylococcus aureus*. 2. Cu-SD loaded GL and GA reduced TNF-α and inhibited IL-6 production.	14982 10114
GD [37]	NA	Intraperitoneal injection of STZ (70 mg/kg) dissolved in 0.1 M sodium citrate buffer (pH 4.5)	A full-thickness skin wound with a diameter of 1.5 cm was created on the back of the rat using a pathological perforator	Male SD rats	Apply 100 μL DC solution (100 mg/mL) to the wound	1. DC could up-regulate the expression of CD31 and VEGFR, and increase the expression levels of Nrf2, HO-1 and NQO-1. 2. Decreased expression of Bax and increased expression of Bcl-2. 3. DC can accelerate wound healing and promote angiogenesis.	NA
HB Liniment [44]	NA	Intraperitoneal injection of STZ (60 mg/kg) dissolved in citrate buffer (pH 4.5)	Two full-thickness wounds with a diameter of 2 cm were created on the back of the rat	SD rats	Low dose and high dose HB liniment were applied to the wound	1. HB can increase the growth of new collagen and promote wound healing. 2. HB decreased MDA and 8-OHdG contents, which indicated that HB linimant inhibited oxidative damage and apoptosis. 3. HB can enhance Nrf2 and its downstream antioxidant genes, thereby reducing oxidative damage.	NA
Icariin [31]		Intraperitoneal injection of STZ(52 mg/kg) dissolved in 0.1 M citrate buffer	An open excision wound of approximately 4 cm^2^ was created in the dorsal thoracolumbar spine of the rat, with a depth of approximately the circular muscle of flesh	Male Wistar rats	Topical application of 0.04% (w/w) icariin ointment was applied to the wound area twice daily	1. Icariin can promote wound healing. 2. Icariin can increase the level of IL-10, increase the expression of CD31, and promote collagen deposition. 3. Icariin can reduce the activity of MMP-2 and MMP-9 soluble.	5318997
*Momordica charantia* ointment [20]	NA	Intravenous injection of STZ (50 mg/kg)	Four full-thickness skin excision wounds were created in the dorsal thoracolumbar region of the rat using a 6-mm puncture biopsy needle	Male SD rats	Apply *momordica charantia* ointment (50 mg) to the wound daily	*Momordica charantia* ointment can improve the wound closure rate, promote the complete epithelization of dermis and epidermis, and promote the uniform arrangement of collagen fibers.	NA
*Nephthea sp.* methanol-methylene chloride extract [17]	NA	Intraperitoneal injection of STZ(60 mg/kg)	A 1 cm diameter perforator was used to create a full-thickness skin wound on the back (scapular region) of the rat	Male Wistar rats	Sterile gauze soaked in the extract was applied topically to the wound and changed twice daily	This extract can improve the wound healing rate. 2. This extract can reduce inflammatory proteins.	NA
PSEO [27]	NA	Intraperitoneal injection of STZ (50 mg/kg)	Incisional wounds of 2 × 2 cm in size and 2 mm in depth were created on the back of the animal using scissors and scalpel	Male Wistar rats	Topical 0.5% Sibirica pine essential oil ointment and 0.5% sibirica pine essential oil gel	1. PSEO can promote wound healing. 2. PSEO can increase the content of HYP in wound tissue. 3. PSEO preparation can enhance the enzyme activity of antioxidant defense system.	NA
*Withania coagulans* extract [28]	NA	Intraperitoneal injection of STZ (70 mg/kg)	Wounds were created on the back of the rat	SD rats	An ointment made from the aqueous alcohol extract of *Withania coagulans*, applied externally to wounds; The aqueous alcoholic extract of *Withania coagulans* was dissolved in distilled water and administered orally once daily at a dose of 500 mg/kg	*Withania coagulans* extract may accelerate wound healing in diabetic rats by increasing collagen synthesis and cell proliferation and enhancing antioxidant capacity, and oral is superior to topical application.	NA
Extract of *Euphorbia hirta* [33]	NA	Single injection alloxan monohydrate (120 mg/kg)	A resection wound with a size of 4.90 cm^2^ and a depth of 2 mm was created	Female Swiss albino mice	Oral administration (100, 200 and 400 mg/kg/day); Topical (5%, 10%) ointment made from ethanol extract of *Euphorbia hirta* (50 mg/kg/day)	1. This extract can improve the wound healing rate, and oral administration is superior to topical application. 2. Oral administration reduced blood glucose levels in diabetic rats. 3. Oral administration of the drug reduced the levels of MDA and NO in plasma, indicating its antioxidant effect. 4. The extract could reduce inflammatory cell infiltration.	NA

ZWP: *Curcuma zedoaria* polysaccharide, NA: Not Available, STZ: Streptozotocin, SD rats: Sprague-Dawley rats, PRP-Exos/ZWP: Complex of platelet-rich plasma exosomes with ZWP, EGCG: Epigallocatechin gallate, ICR mice: Institute of Cancer Research, DM mice: Diabetes mellitus, IL-1β: Interleukin-1beta, TNF-α: Tumor necrosis factor-α, IL-6: Interleukin 6, IGLR: Total iridoid glycoside extract of Lamiophlomis rotata, db/db mice: Diabetes mice, CMC-Na: Sodium carboxyme thyl cellulose, NRF2: Nuclear factor erythroid 2-related factor 2, COX2: Cyclooxygenase-2, KEAP1: Kelch - like ECH - associated protein 1, T.Polium: *Teucrium polium*, HLG: H.lucorum gastropod, AFG: Snail glycosaminoglycan, GelMA: Methacrylic Acid Gel, CGE: *Calvatia gigantea* extract, PE: Pyrolytic palm kernel shell wood vinegar, ACSL4: Long-chain acyl-CoA synthetase 4, GPX4: Glutathione Peroxidase 4, MOL: Extract of *Moringa oleifera*, Col1a1: Collagen type I alpha 1 chain Gene, MCP-1: Monocyte Chemoattractant Protein-1, HA-PF: Hyaluronic Acid-Paeoniflorin, CD31: Platelet endothelial cell adhesion molecule-1, VEGF: Vascular endothelial growth factor, HST: *Hesperetin*, GSH/GSSG: Glutathione/Oxidized Glutathione, MDA: Malondialdehyde, Ast: *Astragalus*, MOL: Extract of *Moringa oleifera*, NAD: Nicotinamide Adenine Dinucleotide, Cur: Curcumin, CNPs@GMs: Curcumin nanoparticles loaded with gelatin microspheres, ROS: Reactive Oxygen Species, BJ Cell: Human Skin Fibroblast Cells, GPX: Glutathione peroxidase, α-SMA: Alpha smooth muscle actin, UA: Ursolic Acid, AVE: Aloe Vera Extract-Loaded Nanofibrous Poly, IL-10: Interleukin-10, TIMP: Tissue inhibitors of metalloproteinases, MMP-2: Matrix Metallopeptidase 2, MMP-9: Matrix metallopeptidase 9, HEA: Hydroalcoholic extract of agave, OAT: Ethanolic extract of *Avena sativa*, KGM: Konjac glucomannan, KER: Human hair proteins, TMP: Tetramethylpyrazine, TMP-HA: TMP-loaded Hyaluronic acid hydrogel, STAT1: Signal transducer and activator of transcription 1, iNOS: Inducible nitric oxide synthase, STAT6: ignal transducer and activator of transcription 6, Arg-1: Arginase-1, C66: (2E,6E)-2,6-bis[2-(trifluoromethyl) benzylidene] cyclohexanone, miR-146a: MIR146A Gene-MicroRNA 146a, IL-8: Interleukin 8, IRAK1: Interleukin 1 receptor associated kinase 1 Gene, NF-κB: Nuclear factor kappa-B, SGAG: Snail glycosaminoglycan, Cur: Curcumin, PCSA: Poly(vinyl alcohol)-chitosan/sodium alginate-Cur, CG: Clerodendrum Glandulosum, AgNP: Clerodendrum glandulosum extract reduced silver nanoparticle, PEG: chitosan-polyethylene glycol, ECM: ExtraCellular Matrix, PvE-3: Earthworm extract, AVE: Aloe Vera Extract-Loaded Nanofibrous Poly, FPG: Fasting plasma glucose, TG: Triglyceride, CHOL: Cholesterol, Apo-B: Apolipoprotein B, Apo-E: Apolipoprotein E, CD68: Cluster of Differentiation 68, CD34: CD34 molecule Gene, HCE: Aqueous-ethanol extract of Horse chestnut, HYP: Hydroxyproline, MMP-1: Matrix Metalloproteinase-1, APN: Apigenin, GGCH-HGs: Apigenin loaded gellan gum-chitosan hydrogels, GL: *Glycyrrhizic acid*, GA: Glycyrrhetinic acid, Cu-SD: A water stable cyclodextrin MOF, α-GLU:α-Glucosidase, GD: *Gynura divaricata(L.)DC*, VEGFR:Vascular endothelial growth factor receptor, HO-1: Heme Oxygenase-1, NQO-1: (NAD(P)H quinone oxidoreductase 1, Bax: BCL-2-associated X protein, Bcl-2: B-cell lymphoma-2, HB:*Huangbai*, 8-OHdG: 8-Hydroxy-2′-deoxyguanosine, PSEO: *Pinus sibirica essential oil*, NO: Nitric Oxid.

**Table 2:** Reports preclinical studies of natural compounds for the treatment of diabetes-related wounds or ulcers.

Chemical compound	Time of execution	Type of study	Number of subjects	Duration of intervention	Methods of Administration	Results
*Phyllanthus niruri* Linn.and *Sida cordifolia* Linn [54].	June 2014 to February 2016	RCT	Ninety subjects were divided into three groups	11 weeks	(experimental group 1) DE group: patients received a decoction made of 7g coarse-powdered *S. cordifolia* root taken twice daily, and a decoction made of 3 g whole-plant *Phyllanthus niruri* powder taken three times daily, all taken with water. (experimental group 2) EX group: patients took two red capsules (each capsule containing 3.6 g coarse powder of *S. cordifolia* root extract) and two green capsules (each capsule containing 1.5 g fine powder of *Phyllanthus niruri* whole plant extract) three times daily, both with water	1. DE group and EX group can improve patients' symptoms (such as pain, tenderness, burning pain, numbness) 2. Both of them can improve the vibration, thermal sensation threshold of patients.
Flaxseed oil omega-3 fatty acids [57]	From April 2016 to July 2016	RCT	Sixty subjects were divided into two groups	12 weeks	Oral 1000 mg linseed oil-3 fatty acid supplement twice daily	1. Flaxseed oil omega-3 fatty acid supplements reduce the length, width, and depth of ulcers. 2. It can reduce insulin concentration, IR, HbA1c and hs-CRP Concentration. 3. It can improve the quantitative insulin sensitivity test index, T-OAC, GSH concentration.
PA-F4 and ON101[47]	November 23, 2012 to 11 May 2020	RCT	236 subjects were divided into two groups	16 weeks	The ON101 dressing was applied twice daily	ON101 can increase the incidence of complete healing, shorten the time required for complete healing, and increase the proportion of patients with ulcer surface area reduction of 50%, with high safety.
*Ageratina pichinchensis* extract [55]	NA	RCT	36 subjects were divided into two groups	24 weeks	Cream made from *Ageratina pichinchensis* extract (5%) was applied topically	*Ageratina pichinchensis* extract was able to shorten the time of wound healing.
YaSP extract [56]	NA	RCT	2 groups with at least 23 participants in each group	12 weeks	Topical YaSP solution (approximately 2 mL for 1 cm^2^ wound area)	1. YaSP extract can improve the healing rate of patients. 2. It can reduce the production of NO in RAW 264.7 macrophage cells induced by LPS, and can remove NO free radicals. 3. It can enhance the migration of human dermal fibroblasts and inhibit the formation of *Staphylococcus epidermidis* biofilm.
Kiwifruit extract [51]	From 2009 to 2010	RCT	40 subjects were divided into two groups	21 days	Kiwifruit extract was overlaid on the ulcer twice a day	1. Kiwifruit extract can promote the reduction of foot ulcer surface area and promote the level of angiogenesis and vascularization. 2. Increase the amount of collagen and granulation tissue to accelerate wound healing.
Honey and olive oil [50]	NA	RCT	45 subjects were divided into 3 groups	1 month	The wound was covered with gauze containing honey and olive oil once a day	Honey and olive oil can improve wound healing, promote wound drainage, and increase the average score of wound healing, but can not reduce the blood glucose of patients.
Qu and OA [46]	From February 2017 to October 2019	RCT	56 subjects were divided into 2 groups	2 months	Nanohydrogels embedded with equimolar doses of Qu and OA were used topically	Qu and OA embedded nanohydrogels were able to significantly shorten the wound healing time without adverse drug reactions.
Olive oil [52]	March 1, 2014 to September 30, 2014	RCT	30 subjects were divided into 2 groups	4 weeks	Refined olive oil was applied to the ulcer surface and then covered with gauze soaked in olive oil	1. Olive oil can improve the degree of ulcers, surrounding tissues and the overall state of ulcers, promote the reduction of ulcer area and depth, and increase the proportion of complete healing without adverse reactions. 2. Olive oil does not promote ulcer drainage.
*Centella asiatica* extract [53]	June 2008 to September 2009	RCT	200 subjects were divided into 2 groups	21 days	Two *Centella asiatica* extract capsules were taken orally three times a day	Oral *Centella asiatica* extract can promote wound contraction in diabetic patients, shorten the contraction time, and has good safety. However, it could not promote the formation of granulation tissue.
Latex of P1G10[58]	From August 2012 to October 2016	RCT	Fifty people. One group of 23 and one group of 27	16 weeks	0.1% P1G10 was used, formulated in Polawax. Dressings containing P1G10 were applied to the patient's ulcer three times a week	P1G10 treatment can improve the complete wound healing rate.
*Hypericum perforatum* and *Azadirachta indica* [49]	From June 25, 2012 to December 20, 2012	CSR	A 67-year-old woman	From July 2, 2012 to December 20, 2012	A mixture of plant extracts was applied to the gauze and applied to the ulcer	Treatment based on this plant extract can improve ulcer condition and reduce HbA1c in patients with diabetic foot.
*Hypericum perforatum* and *Azadirachta indica* [48]	Treatment was started on the left foot on 5 March 2012 and on the right foot on 13 March 2012.	CSR	A 72-year-old man	4 months	A mixture of plant extracts was applied to the gauze and applied to the ulcer	Treatment with the plant extract was able to promote the epithelialization of granulation tissue and wound healing, improving the ulcer condition of the patient's feet.

RCT: andomized controlled trial, IR: Insulin resistance, hs-CRP: hypersensitive C-reactive protein, T-OAC: Total Antioxidant Capacity, PA-F4:Plectranthus amboinicus extract, ON101: *Centella asiatica* extract(S1), YaSP: *Ya-Samarn-Phlae*, NO: Nitric Oxide, LPS: Lipopolysaccharide, Qu: Quercetin, OA: Oleic acid, P1G10: Latex of *Vasconcellea cundinamarcensis*, CSR: Clinical Study Report.

**Fig. 2:**
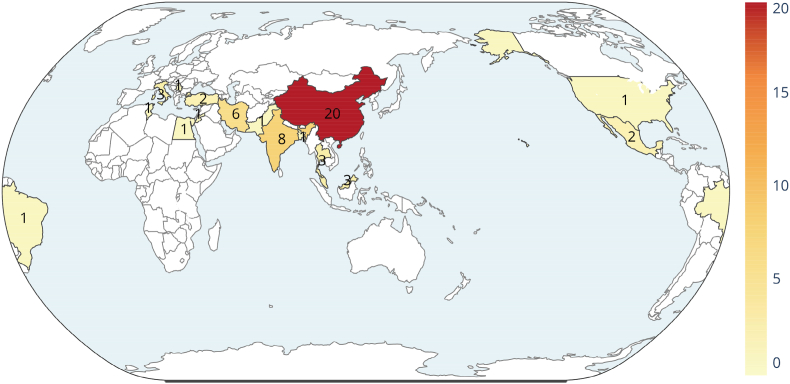
World distribution of countries of corresponding or first authors for preclinical or clinical studies.

**Fig. 3:**
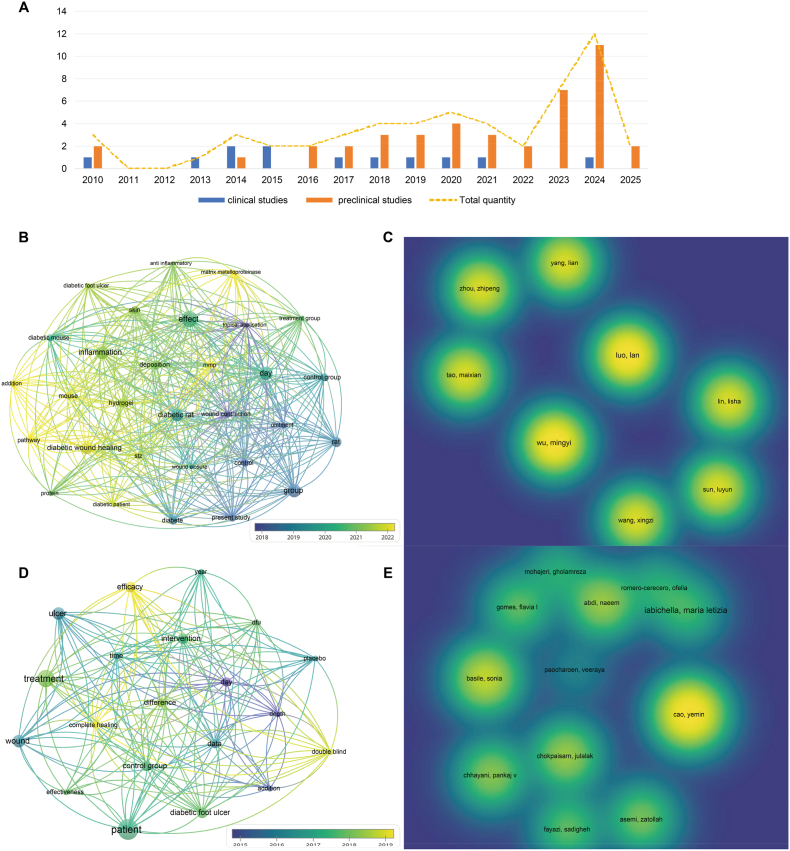
Bibliographic analysis for the current status and development trends of the included literature. A: Literature of publication numbers by year; B: Word cloud map of preclinical research abstracts; C: Core authors of preclinical research; D: Word cloud map of clinical research abstracts; E: Core authors of clinical research.

**Fig. 4:**
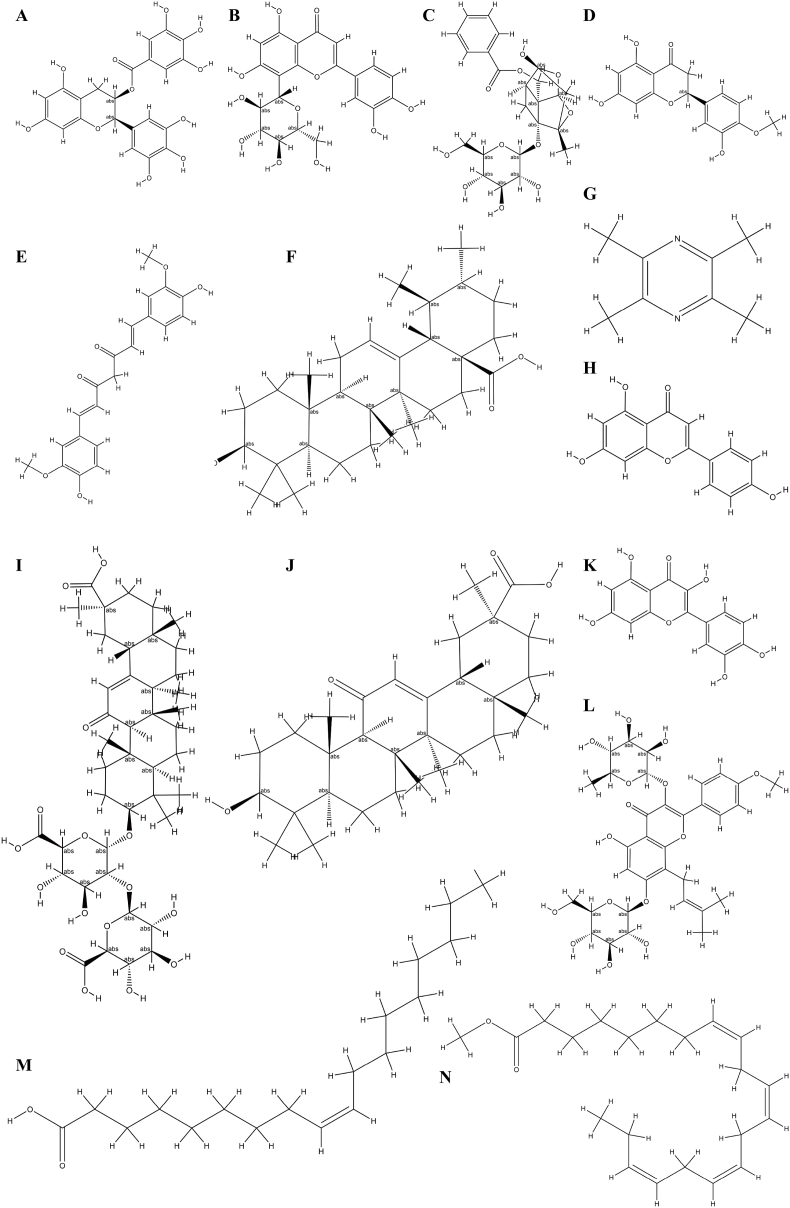
Compound structural formula. A: *Epigallocatechin gallate*; B: Orientin; C: Hyaluronic Acid - Paeoniflorin; D: *Hesperetin*; E: Curcumin; F: Ursolic Acid; G: Tetramethylpyrazine; H: Apigenin; I: *Glycyrrhizic acid*; J: *Glycyrrhetinic acid*; K: Icariin; L: Quercetin; M: Oleic acid; N: Omega-3.

The countries of the corresponding or first author of the included papers were summarized in Fig. 2. The distribution of paper was as follows: Tunisia: one; Egypt: one; India: eight; Iran: six; China: 20; Thailand: three; Pakistan: one; Malaysia: three; Jordan: one; Bangladesh: one; Turkey: two; Italy: three; Serbia: one; Mexico: two; United States: one; Brazil: one. In addition, the keyword and author analysis was performed by VOSviewer (1.6.19) in a Java environment. Excel 2019 was used to statistically describe the annual publication volume of literature. The study summarizes the current status and development trends of the included literature in Fig. 3. This includes literature of publication numbers by year, word cloud map from the abstracts, and core author analysis. The chemical structures of natural compounds were summarized in Fig. 4, using Photoshop.

### 3.1 Polyphenols

**Fig. 5:**
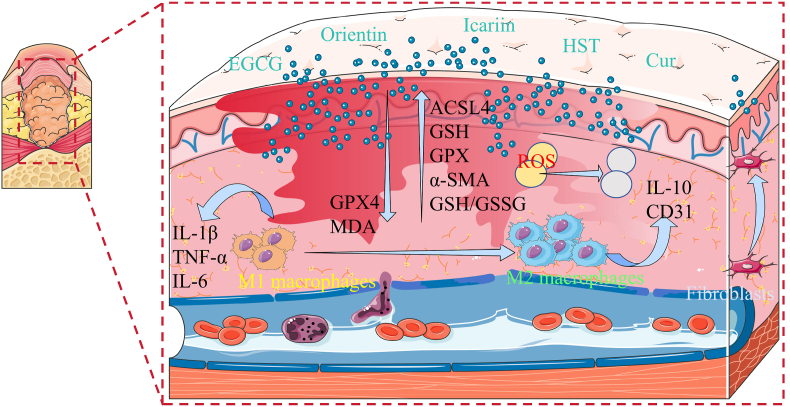
Diagram of the mechanism of action of polyphenolic natural compounds on diabetes-related wounds or ulcers.

Polyphenolic compounds include flavonoids, tannins, phenolic acids and anthocyanins. In addition, a review summarized the significant therapeutic potential of edible flavonoids produced by plants themselves [59]. These compounds have the effects of anti-oxidation, lowering blood lipids, enhancing immunity, preventing arteriosclerosis and thrombosis. It is commonly found in tea, grapes and various herbs. The mechanism of polyphenolic compounds in diabetic-related wounds or ulcers was shown in Fig. 5.

Through the inhibition of Notch signaling pathway, *Epigallocatechin gallate* (EGCG) can reduce levels of inflammatory factors such as Interleukin-1beta (IL-1β), Tumor necrosis factor-α (TNF-α), and Interleukin 6 (IL-6), decrease macrophage accumulation in diabetic mice wounds, and improve wound healing [19]. Orientin enhance angiogenesis and wound healing by upregulating Long-chain acyl-CoA synthetase 4 (ACSL4), downregulating Glutathione peroxidase (GPX4), and reducing the accumulation of mitochondrial reactive oxygen species [40].

Following hesperetin treatment, there was a significantly increase in the Glutathione/Oxidized Glutathione (GSH/GSSG) ratio, and the therapeutic effect was dependent on the dose. In addition, hesperetin effectively inhibited the increase of malondialdehyde (MDA) level after erastin stimulation, promoted the recovery of cell viability and morphology, and offset the inhibition of angiogenesis induced by erastin. It also has the potential to promote wound healing by inhibiting ferroptosis and restoring mitochondrial function [41]. Curcumin promoted the transformation of M1 macrophages into M2 macrophages, increased glutathione (GSH) and glutathione peroxidase (GPX) levels, reduced reactive oxygen species (ROS) levels. It can simultaneously increase the expression of α-smooth muscle actin (α-SMA), promoted fibroblast migration and granulation tissue formation, and improved wound contraction rate [14,23]. Apigenin contributes to wound contraction, increases the weight of granulomas tissue, and promote the healing of diabetic and normal wounds [29]. By increasing Interleukin-10 (IL-10) levels and Platelet endothelial cell adhesion molecule-1 (CD31) expression, icariin reduces matrix metallopeptidase 2 (MMP-2) and matrix metallopeptidase 9 (MMP-9) activity, promotes collagen deposition, and promotes the healing of wounds [31]. In addition, clinical studies have shown that nanohydrogel embedded with quercetin and oleic acid can significantly shorten wound healing time without adverse reactions [46].

### 3.2 Terpenoids

**Fig. 6:**
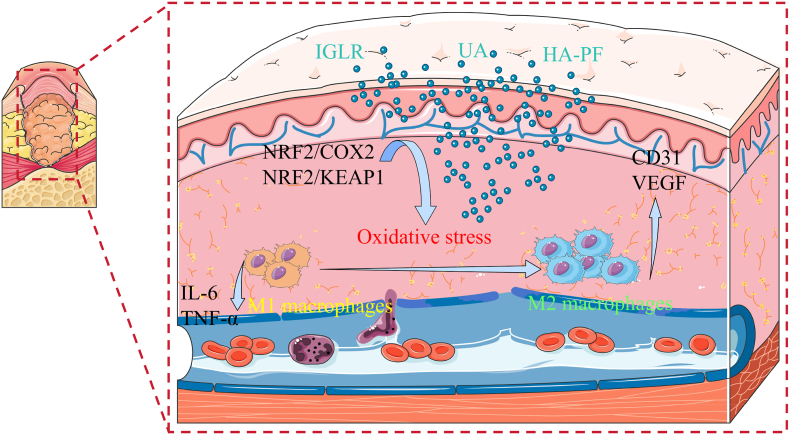
Diagram of the mechanism of action of terpenoid natural compounds on diabetes-related wounds or ulcers.

Terpenoids include monoterpenes, sesquiterpenes, iridoids, diterpenes. Terpenoids are commonly found in herbs and plants including peony, hawthorn, mint, gardenia, rose oil, and turpentine. The mechanism of terpenoids compounds in diabetic-related wounds or ulcers was shown in Fig. 6.

Total iridoid glycoside extract of Lamiophlomis rotata (IGLR) inhibit oxidative stress and inflammation through nuclear factor erythroid 2-related factor 2/cyclooxygenase-2 (NRF2/COX2) signaling pathway. They also inhibit oxidative stress through nuclear factor erythroid 2-related factor 2/Kelch like ECH associated protein 1 (NRF2/KEAP1) signaling pathway and paracrine mechanism, which induces fibroblasts and promoting wound healing [15]. Paeoniflorin hydrogel can improve wound healing by reducing inflammatory, improving epidermal hyperplasia and collagen distribution, increasing the number of M2 macrophages and new blood vessels, and enhancing the expression of CD31 and vascular endothelial growth factor (VEGF) [39]. By reducing IL-6 and TNF-α levels, ursolic acid (UA) promotes the transformation of macrophage phenotype from M1 to M2, increases the collagen content of the wound and the coverage area of CD31-positive tissue, thereby improving the wound closure rate [24].

### 3.3 Polysaccharides

Polysaccharides include starch, glycogen, cellulose, and glycosaminoglycans. Polysaccharide are commonly present in plants, animals, and microorganisms, such as *Curcuma zedoaria* and *Snails*. In diabetic rats, ZWP can improve wound contraction rate, epidermal thickness, collagen synthesis and deposition [38]. By increase vascular density and number of new blood vessels, glycosaminoglycans can accelerate wound healing, while also reducing early swelling and inflammatory reaction to injury [22,36,45].

### 3.4 Saponins

Saponins include steroidal saponins and triterpenoid saponins, which are widely found in plants such as *Licorice*, *Ginseng*, *Anemarrhena asphodeloides*, *Platycodon grandiflorum*, and *Bupleurum*. In-vitro studies, glycyrrhizic acid and glycyrrhetinic acid can inhibit α-Glucosidase (α-GLU) activity, reduce TNF-α and IL-6 levels, inhibit the number of *Escherichia coli* and *Staphylococcus aureus*, and promote wound healing [43].

### 3.5 Organic acids

Organic acids include aromatic organic acids, aliphatic organic acids and terpenoid organic acids. These compounds are commonly present in the leaves, roots, and fruits of plants such as *Black plum*, *Schisandra chinensis*, *and Raspberries*. The effects of organic acids include blood vessel dilation, improved microcirculation, and anti-inflammatory, antibacterial, antiviral, and anti-tumor actions. Omega-3 fatty acids from flaxseed oil promote diabetic wound healing by reducing the level of high-sensitivity C-reactive protein (hs-CRP), Total Antioxidant Capacity (T-OAC), and GSH and reducing the length, width, and depth of ulcers [57].

### 3.6 Other compounds

Tetramethylpyrazine (TMP) promotes wound healing by promoting the transformation of M1 macrophages to M2 macrophages, downregulating Signal transducer and activator of transcription 1 (STAT1) activity and Inducible nitric oxide synthase (iNOS) expression, and upregulating Signal transducer and activator of transcription 6 (STAT6) activity and the expression of Arginase-1 (Arg-1), CD31, VEGF, α-SMA and Collagen type I alpha 1 chain Gene (COL1a1) [11]. In diabetic mice, (2E,6E)-2,6-bis[2-(trifluoromethyl) benzylidene] cyclohexanone (C66) increased the re-epithelialization, angiogenesis and percentage of proliferating cells in wounds, and improved the healing rate of wounds. These effects were related to increasing the level of MIR146A Gene-MicroRNA 146a (miR-146a), inhibiting the expression of TNF-α, IL-6, and Interleukin 8 (IL-8), and inhibiting the activity of Nuclear factor kappa-B (NF-κB) by targeting Interleukin 1 receptor associated kinase 1 Gene (IRAK1) [18]. *Pinus sibirica* essential oil (PSEO) promote wound healing by increasing the content of Hydroxyproline (HYP) in wound tissue and enhancing the activity of antioxidant enzymes [27]. Olive oil improve the severity of ulcers, promote the reduction of ulcer area and depth, and increase the proportion of complete healing [52].

### 3.7 Herbs or plants

There was herbal or plant formulations used for treating diabetes-related wounds or ulcers, although the ingredients have not been determined. *Astragalus* (Ast) gum preparation can improve wound contraction rate by promoting angiogenesis and inhibiting *Staphylococcus aureus* and *Escherichia coli* [7]. *Gynura divaricata*(L.)DC (GD) accelerate wound healing and promote angiogenesis by upregulating the expression of CD31, Vascular endothelial growth factor receptor (VEGFR), B-cell lymphoma-2 (Bcl-2), NRF2, Heme Oxygenase-1 (HO-1), and NAD(P)H quinone oxidoreductase 1 (NQO-1), along with the downregulation of BCL-2-associated X protein (Bax) [37]. In diabetic rats, *Huangbai* (HB) linimin increases collagen synthesis and promote wound healing. It decreases MDA and 8-Hydroxy-2′-deoxyguanosine (8-OHdG), increases NRF2 and antioxidant gene expression, and reduces oxidative damage [44]. *Bitter melon* ointment improves the speed of wound healing by promoting complete epithelialization of the dermis and epidermis and promoting uniform arrangement of collagen fibers [20]. In clinical studies, *Phyllanthus niruri Linn.* and *Sida cordifolia Linn* can alleviate symptoms including pain, tenderness, burning, and numbness, while also raising sensory thresholds for vibration, cold, and heat [54]. Olive oil can improve wound healing, promote wound drainage, and increase the average score of wound healing [50].

### 3.8 The extracts from plant or herbal

In some studies, the components or compounds of the extracts have yet to be identified. The extracts of *Teucrium polium* [14] (*T. polium*), *Calvatia gigantea* [12] (CG) and Pyrolytic palm kernel shell wood vinegar [32] (PE) can improve the wound healing rate. Notably, CG extract can improve the wound healing rate, collagen fiber density, anti-inflammatory cytokines levels, and upregulate the number of M2 macrophages [25]. Extract of *Moringa oleifera* (MOL) extract enhance wound healing and contraction rates by upregulating the expression of COL1a1, IL-6, and Monocyte Chemoattractant Protein-1 (MCP-1), promoting collagen deposition, and shortening epithelialization time. In-vitro, MOL extract exhibit antibacterial properties against *Staphylococcus aureus*, *Pseudomonas aeruginosa*, and *Escherichia coli* [10,26]. *Rosemary* extract can increase the weight of granulation tissue and improve wound contraction rate [4]. Aloe Vera Extract-Loaded Nanofibrous Poly (AVE) impregnated nanofiber pads increased the percentage of wound contraction and had reduced inflammatory cytokine levels and antimicrobial effects [16]. The hydrogel made from banyan tree and onion extracts can increase IL-10. Tissue inhibitors of metalloproteinases (TIMP) levels and wound contraction rate, reduce TNF-α, IL-6, MMP-2, and MMP-9 levels [42]. Hydroalcoholic extract of *Agave* [6] (HEA) and Ethanolic extract of *Avena sativa* [34] (OAT) can promote wound tissue repair. In addition, OAT also increases collagen deposition and fibroblast numbers, and inhibits the number of *Staphylococcus aureus*. Aqueous-ethanol extract of *Horse chestnut* (HCE) promotes wound healing by increasing HYP level and Matrix Metalloproteinase-1 (MMP-1) gene expression, and reducing MDA level [6]. *Earthworm* extract (PvE-3) can accelerate wound healing and promote neovascularization [35]. *Bitter melon* extract can prevent granulation tissue degeneration and increase the thickness of granulation tissue. Its mechanism may be related to increasing the levels of Cluster of Differentiation 68 (CD68) positive cells, myofibroblasts, VEGF, CD34 molecule Gene (CD34) positive cells and blood vessel density in granulation tissue [30]. The extract from *Ginkgo biloba* contributes to epidermal integrity, dermal regeneration, and collagen organization, while enhancing epithelialization scores [9]. The use of *Ashwagandha* extract in diabetic rats can improve wound healing by increasing collagen synthesis, enhancing cell proliferation, and boosting antioxidant capacity, with oral administration being more beneficial than topical application [28]. Applying *Euphorbia* extract topically can decrease the infiltration of inflammatory cells and enhance the rate of wound healing [33]. In clinical studies, extract of *Centella asiatica* extract(S1) [47] (ON101), *Ageratina pichinchensis* [55], *Centella asiatica* [53], and Latex of *Vasconcellea cundinamarcensis* [58] (P1G10) can increase the incidence of complete healing and shorten the time required for complete healing. The healing rate of patients was improved by *Ya-Samarn-Phlae* (YaSP) extract, which enhanced fibroblast migration and prevented the formation of *Staphylococcus* epidermidis biofilm [56]. *Kiwifruit* extract promotes the level of angiogenesis and vascularization, increases the amount of collagen and granulation tissue, promotes the reduction of the surface area of foot ulcers, and accelerates wound healing [51]. Extracts from *Hypericum perforatum* and *Azadirachta indica* can enhance the ulcer condition and promote the formation of granulation tissue and epithelial healing [48,49].

## 4 Discussion

This review synthesizes evidence from 55 preclinical and clinical studies supporting the potential of natural compounds in promoting the healing of diabetes-related wounds or ulcers. Despite promising results, several limitations must be acknowledged. The high heterogeneity among included studies—particularly in terms of compound sources, dosages, administration routes, and outcome measures—limits the ability to draw definitive conclusions. Most clinical trials were small in scale and short in duration, and only 11 were randomized controlled trials focused on diabetic foot ulcers. Moreover, many preclinical studies relied on rodent models, which may not fully recapitulate the complex pathophysiology of human diabetic wounds, especially in terms of chronicity and comorbidity burden.

**Fig. 7:**
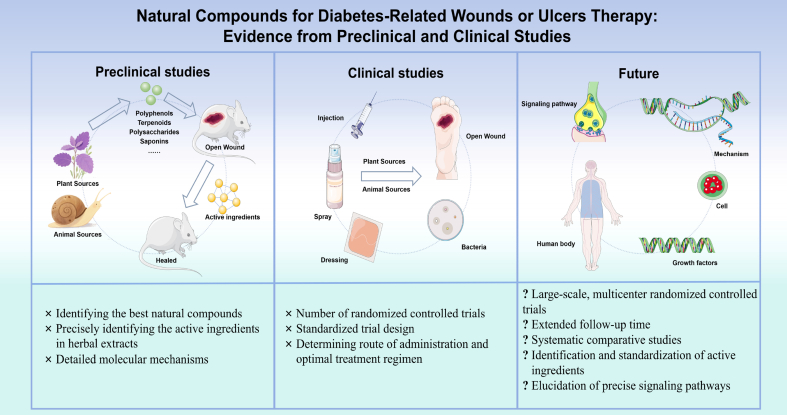
Schematic diagram of the limitations and future directions of the research field.

Future research should prioritize well-designed, large-scale, multicenter randomized controlled trials with standardized protocols for compound preparation, dosage, and treatment duration. Long-term follow-up is essential to assess recurrence rates and long-term safety. Mechanistic studies are needed to elucidate precise molecular targets and signaling pathways, such as those involving macrophage polarization, oxidative stress, and angiogenesis. It can further integrate cutting-edge technologies such as artificial intelligence to deepen understanding. For example, AI-enhanced network pharmacology provides a more powerful tool for analyzing the multi-component-multi-target-multi-pathway interactions of complex traditional Chinese medicine prescriptions [60]. In addition, large language models have shown great potential in mining and analyzing massive amounts of unstructured traditional medical texts and clinical medical records. They can systematically summarize medication patterns and provide novel, traditional wisdom-based clues for modern drug development [61]. Additionally, the development of advanced delivery systems—such as hydrogels and nanoparticle-based carriers—should be optimized to enhance bioavailability and site-specific release. Further exploration of synergistic effects between natural compounds and conventional therapies may also provide novel combination strategies. Ultimately, translating these preclinical insights into clinically viable treatments will require rigorous validation and standardization to ensure efficacy, safety, and reproducibility. More clearly illustrates the limitations and future directions of this research field in Fig. 7.

## 5 Conclusions

This review consolidates evidence from 55 preclinical and clinical studies supporting the therapeutic potential of natural compounds in managing diabetic skin ulcers. Key compounds such as ursolic acid, paeoniflorin, and *Curcuma zedoaria* polysaccharide (ZWP) demonstrate promising efficacy through mechanisms including anti-inflammatory action, promotion of angiogenesis, macrophage polarization, and enhanced collagen deposition.

Despite encouraging results, clinical application remains challenged by a lack of standardized dosing, administration routes, and formulation strategies. Hydrogels and nano-delivery systems show potential as effective local carriers, yet their optimal design requires further investigation.

Future studies should focus on well-designed randomized controlled trials to validate efficacy, optimize delivery systems, and establish clear treatment protocols. With continued research, natural compounds are poised to become valuable additions to the therapeutic arsenal against diabetic wounds.

## Authors' contributions

Jia Teng: Conceptualization, Methodology, Formal analysis, Writing - original draft, review & editing. Guanchi Yan: Conceptualization, Methodology, Formal analysis, Writing - original draft. Ming Yang: Resources. Shuangyue Wang: Formal analysis. Yuezhu Zhao: Supervision. Yanyan Wang: Resources, Formal analysis, Supervision. Jia Mi: Resources, Formal analysis, Supervision. Jia Teng and Guanchi Yan contributed equally to this manuscript. Correspondence should be addressed to Yanyan Wang and Jia Mi. All authors contributed to the article and approved the submitted version.

## Funding

The research was founded by the 10.13039/501100013061Jilin Scientific and Technological Development Program (YDZJ202401120ZYTS), Jilin Province Health Science and Technology Capacity Improvement Project (2024A080).

## Declaration of competing interest

The authors declare that the research was conducted without any potential conflicts of interest.

## Data Availability

All data related to the research are included in the article and Supplementary Material. The included trials were published on open access websites and databases. If necessary, contact the first author for information. The review did not require prior ethical approval and consent from the participants because it only involved previously published RCT studies.

## References

[1] (2025). IDF diabetes atlas.

[2] McDermott K., Fang M., Boulton A.J.M., Selvin E., Hicks C.W. (2023). Etiology, epidemiology, and disparities in the burden of diabetic foot ulcers. Diabetes Care.

[3] (2015). Regranex.

[4] Abu-Al-Basal M.A. (2010). Healing potential of Rosmarinus officinalis L. on full-thickness excision cutaneous wounds in alloxan-induced-diabetic BALB/c mice. J Ethnopharmacol.

[5] Aguilar-Guadarrama A.B., Diaz-Roman M.A., Osorio-Garcia M., Deciga-Campos M., Rios M.Y. (2024). Chemical constituents from Agave applanata and its antihyperglycemic, anti-inflammatory, and antimicrobial activities associated with its tissue repair capability. Planta Med.

[6] Aksoy H., Çevik Ö., Şen A., Göğer F., Şekerler T., Şener A. (2019). Effect of Horse-chestnut seed extract on matrix metalloproteinase-1 and -9 during diabetic wound healing. J Food Biochem.

[7] Amiri Z., Molavi A.M., Amani A., Moqadam K.H., Vatanchian M., Hashemi S.A., Oroojalian F. (2024). Fabrication, characterization and wound-healing properties of core-shell SF@chitosan/ZnO/Astragalus arbusculinus gum nanofibers. Nanomedicine.

[8] Babu S.N., Govindarajan S., Noor A. (2021). Aloe vera and its two bioactive constituents in alleviation of diabetes -proteomic & mechanistic insights. J Ethnopharmacol.

[9] Bardaa S., Makni K., Boudaouara O., Bardaa T., Ktari N., Hachicha S., Ben Salah R., Kallel R., Sahnoun Z., Boufi S. (2021). Development and evaluation of the wound healing effect of a novel topical cream formula based on Ginkgo biloba extract on wounds in diabetic rats. BioMed Res Int.

[10] Chin C.-Y., Ng P.-Y., Ng S.-F. (2018). Moringa oleifera standardised aqueous leaf extract-loaded hydrocolloid film dressing: in vivo dermal safety and wound healing evaluation in STZ/HFD diabetic rat model. Drug Deliv Transl Res.

[11] Chu D., Chen J., Liu X., Liao A., Song X., Li Y., Yang L., Chen Z., Yu Z., Guo J. (2023). A tetramethylpyrazine-loaded hyaluronic acid-based hydrogel modulates macrophage polarization for promoting wound recovery in diabetic mice. Int J Biol Macromol.

[12] Ding X., Yang C., Li Y., He T., Xu Y., Cheng X., Song J., Xue N., Min W., Feng W., Zhao H., Dong J., Liu P., Wang Y., Chen J. (2024). Reshaped commensal wound microbiome via topical application of Calvatia gigantea extract contributes to faster diabetic wound healing. Burns Trauma.

[13] Fallah Huseini H., Abdolghaffari A.H., Ahwazi M., Jasemi E., Yaghoobi M., Ziaee M. (2020). Topical application of teucrium polium can improve wound healing in diabetic rats. Int J Low Extrem Wounds.

[14] Fan X., Huang J., Zhang W., Su Z., Li J., Wu Z., Zhang P. (2024). A multifunctional, tough, stretchable, and transparent curcumin hydrogel with potent antimicrobial, antioxidative, anti-inflammatory, and angiogenesis capabilities for diabetic wound healing. ACS Appl Mater Interfaces.

[15] Geng X., Wang Y., Li H., Song L., Luo C., Gu X., Zhong H., Chen H., Chen X., Wang J., Pan Z. (2024). Total iridoid glycoside extract of Lamiophlomis rotata (Benth) Kudo accelerates diabetic wound healing by the NRF2/COX2 axis. Chin Med.

[16] Ghosh A., Saha K., Bhattacharya T., Sarkar S., Sengupta D., Maiti A., Ghoshal D., Dey S., Chattopadhyay D. (2024). Electrospun cerium oxide nanoparticle/aloe vera extract-loaded nanofibrous Poly(Ethylene Oxide)/Polyurethane mats as diabetic wound dressings. ACS Appl Bio Mater.

[17] Hassan N.H., El-Hawary S.S., Emam M., Rabeh M.A., Tantawy M.A., Seif M., Abd-Elal R.M.A., Bringmann G., Abdelmohsen U.R., Selim N.M. (2023). Pectin nanoparticle-loaded soft coral nephthea sp. extract as in situ gel enhances chronic wound healing: in vitro, in vivo, and in silico studies. Pharmaceuticals.

[18] Huang J., Fu J., Liu B., Wang R., You T. (2020). A synthetic curcuminoid analog, (2E,6E)-2,6-bis(2-(trifluoromethyl)benzylidene)cyclohexanone, ameliorates impaired wound healing in streptozotocin-induced diabetic mice by increasing miR-146a. Molecules.

[19] Huang Y.W., Zhu Q.Q., Yang X.Y., Xu H.H., Sun B., Wang X.J., Sheng J. (2019). Wound healing can be improved by (-)-epigallocatechin gallate through targeting notch in streptozotocin-induced diabetic mice. FASEB J.

[20] Hussan F., Teoh S.L., Muhamad N., Mazlan M., Latiff A.A. (2014). Momordica charantia ointment accelerates diabetic wound healing and enhances transforming growth factor-β expression. J Wound Care.

[21] Ili P., Sari F. (2023). Egg yolk oil accelerates wound healing in streptozotocin induced diabetic rats. Biotech Histochem.

[22] Li Y., Wang X., Chen J., Sun L., Pu D., Lin L., Luo L., Gong X., Pu J., Wu M. (2025). Structural analysis and accelerating wound healing function of a novel galactosylated glycosaminoglycan from the snail Helix lucorum. Carbohydr Polym.

[23] Liu J., Chen Z., Wang J., Li R., Li T., Chang M., Yan F., Wang Y. (2018). Encapsulation of curcumin nanoparticles with MMP9-Responsive and thermos-sensitive hydrogel improves diabetic wound healing. ACS Appl Mater Interfaces.

[24] Lv H., Zhao M., Li Y., Li K., Chen S., Zhao W., Wu S., Han Y. (2022). Electrospun chitosan-polyvinyl alcohol nanofiber dressings loaded with bioactive ursolic acid promoting diabetic wound healing. Nanomaterials.

[25] Majie A., Saha R., Sarkar A., Bhowmik R., Karmakar S., Sharma V., Deokar K., Haque A.U., Tripathy S.S., Sarkar B. (2024). A novel chitosan-PEG hydrogel embedded with in situ silver nanoparticles of Clerodendrum glandulosum lindl. extract: evaluation of its in vivo diabetic wound healing properties using an image-guided machine learning model. Biomater Sci.

[26] Muhammad A.A., Arulselvan P., Cheah P.S., Abas F., Fakurazi S. (2016). Evaluation of wound healing properties of bioactive aqueous fraction from Moringa oleifera lam on experimentally induced diabetic animal model. Drug Des Dev Ther.

[27] Nikolic M., Andjic M., Bradic J., Kocovic A., Tomovic M., Samanovic A.M., Jakovljevic V., Veselinovic M., Capo I., Krstonosic V., Kladar N., Petrovic A. (2023). Topical application of Siberian pine essential oil formulations enhance diabetic wound healing. Pharmaceutics.

[28] Prasad S.K., Kumar R., Patel D.K., Hemalatha S. (2010). Wound healing activity of Withania coagulans in streptozotocin-induced diabetic rats. Pharm Biol.

[29] Shukla R., Kashaw S.K., Jain A.P., Lodhi S. (2016). Fabrication of apigenin loaded gellan gum–chitosan hydrogels (GGCH-HGs) for effective diabetic wound healing. Int J Biol Macromol.

[30] Singh R., Garcia-Gomez I., Gudehithlu K.P., Singh A.K. (2017). Bitter melon extract promotes granulation tissue growth and angiogenesis in the diabetic wound. Adv Skin Wound Care.

[31] Singh W.R., Sharma A., Devi H.S., Bhatia A., Patel M.R., Kumar D. (2022). Icariin improves cutaneous wound healing in streptozotocin-induced diabetic rats. J Tissue Viability.

[32] Theapparat Y., Khongthong S., Roekngam N., Suwandecha T., Nopparat J., Faroongsarng D. (2024). Pyroligneous extract, a biomaterial derived from pyrolytic palm kernel shell wood vinegar, as a novel diabetic wound healing aid: an animal study. Drug Dev Ind Pharm.

[33] Tuhin R.H., Begum M.M., Rahman M.S., Karim R., Begum T., Ahmed S.U., Mostofa R., Hossain A., Abdel-Daim M., Begum R. (2017). Wound healing effect of Euphorbia hirta linn. (Euphorbiaceae) in alloxan induced diabetic rats. BMC Compl Alternative Med.

[34] Veerasubramanian P.K., Thangavel P., Kannan R., Chakraborty S., Ramachandran B., Suguna L., Muthuvijayan V. (2018). An investigation of konjac glucomannan-keratin hydrogel scaffold loaded with Avena sativa extracts for diabetic wound healing. Colloids Surf B Biointerfaces.

[35] Wang W., Ye J., Guo Z., Ma Y., Yang Q., Zhong W., Du S., Bai J. (2023). A novel glycoprotein from earthworm extract PvE-3: insights of their characteristics for promoting diabetic wound healing and attenuating methylglyoxal-induced cell damage. Int J Biol Macromol.

[36] Wu Y., Zhou Z., Luo L., Tao M., Chang X., Yang L., Huang X., Hu L., Wu M. (2020). A non-anticoagulant heparin-like snail glycosaminoglycan promotes healing of diabetic wound. Carbohydr Polym.

[37] Xu C., Hu L., Zeng J., Wu A., Deng S., Zhao Z., Geng K., Luo J., Wang L., Zhou X., Huang W., Long Y., Song J., Zheng S., Wu J., Chen Q. (2024). Gynura divaricata (L.) DC. promotes diabetic wound healing by activating Nrf2 signaling in diabetic rats. J Ethnopharmacol.

[38] Xu N., Wang L., Guan J., Tang C., He N., Zhang W., Fu S. (2018). Wound healing effects of a Curcuma zedoaria polysaccharide with platelet-rich plasma exosomes assembled on chitosan/silk hydrogel sponge in a diabetic rat model. Int J Biol Macromol.

[39] Yang H., Song L., Sun B., Chu D., Yang L., Li M., Li H., Dai Y., Yu Z., Guo J. (2021). Modulation of macrophages by a paeoniflorin-loaded hyaluronic acid-based hydrogel promotes diabetic wound healing. Mater Today Bio.

[40] Yang J.y., Zhuang C., Lin Y.z., Yu Y.t., Zhou C.c., Zhang C.y., Zhu Z.t., Qian C.j., Zhou Y.n., Zheng W.h., Zhao Y., Jin C., Wu Z.y. (2024). Orientin promotes diabetic wounds healing by suppressing ferroptosis via activation of the Nrf2/GPX4 pathway. Food Sci Nutr.

[41] Yu X., Liu Z., Yu Y., Qian C., Lin Y., Jin S., Wu L., Li S. (2024). Hesperetin promotes diabetic wound healing by inhibiting ferroptosis through the activation of SIRT3. Phytother Res.

[42] Zahoor S., Tahir H.M., Ali S., Ali A., Muzamil A., Murtaza Z., Zahoor N. (2023). Diabetic wound healing potential of silk sericin protein based hydrogels enriched with plant extracts. Int J Biol Macromol.

[43] Zhan M., Zhou D., Lei L., Zhu J., Khan M.Z.H., Liu X., Ma F. (2025). Glycyrrhizic acid and glycyrrhetinic acid loaded cyclodextrin MOFs with enhanced antibacterial and anti-inflammatory effects for accelerating diabetic wound healing. Colloids Surf B Biointerfaces.

[44] Zhang J., Zhou R., Xiang C., Jia Q., Wu H., Yang H. (2020). Huangbai liniment accelerated wound healing by activating Nrf2 signaling in diabetes. Oxid Med Cell Longev.

[45] Zhou Z., Deng T., Tao M., Lin L., Sun L., Song X., Gao D., Li J., Wang Z., Wang X., Li J., Jiang Z., Luo L., Yang L., Wu M. (2023). Snail-inspired AFG/GelMA hydrogel accelerates diabetic wound healing via inflammatory cytokines suppression and macrophage polarization. Biomaterials.

[46] Gallelli G., Cione E., Serra R., Leo A., Citraro R., Matricardi P., Di Meo C., Bisceglia F., Caroleo M.C., Basile S., Gallelli L. (2020). Nano-hydrogel embedded with quercetin and oleic acid as a new formulation in the treatment of diabetic foot ulcer: a pilot study. Int Wound J.

[47] Huang Y.Y., Lin C.W., Cheng N.C., Cazzell S.M., Chen H.H., Huang K.F., Tung K.Y., Huang H.L., Lin P.Y., Perng C.K., Shi B., Liu C., Ma Y., Cao Y., Li Y., Xue Y., Yan L., Li Q., Ning G., Chang S.C. (2021). Effect of a novel macrophage-regulating drug on wound healing in patients with diabetic foot ulcers: a randomized clinical trial. JAMA Netw Open.

[48] Iabichella M.L. (2013). The use of an extract of Hypericum perforatum and Azadirachta indica in advanced diabetic foot: an unexpected outcome. BMJ Case Rep.

[49] Iabichella M.L., Caruso C., Lugli M. (2014). The use of an extract of Hypericum perforatum and Azadirachta indica in a neuropathic patient with advanced diabetic foot. BMJ Case Rep.

[50] Karimi Z., Behnammoghadam M., Rafiei H., Abdi N., Zoladl M., Talebianpoor M.S., Arya A., Khastavaneh M. (2019). Impact of olive oil and honey on healing of diabetic foot: a randomized controlled trial. Clin Cosmet Invest Dermatol.

[51] Mohajeri G., Safaee M., Sanei M.H. (2014). Effects of topical Kiwifruit on healing of neuropathic diabetic foot ulcer. J Res Med Sci.

[52] Nasiri M., Fayazi S., Jahani S., Yazdanpanah L., Haghighizadeh M.H. (2015). The effect of topical olive oil on the healing of foot ulcer in patients with type 2 diabetes: a double-blind randomized clinical trial study in Iran. J Diabetes Metab Disord.

[53] Paocharoen V. (2010). The efficacy and side effects of oral Centella asiatica extract for wound healing promotion in diabetic wound patients. J Med Assoc Thail.

[54] Patel M.V., Patel M.M., Patel K.B., Chhayani P.V., Mittwede M., Scheidbach D., Gupta S.N. (2022). A randomized placebo-compared study on the efficacy of classical ayurvedic pharmaceutical form versus aqueous alcoholic extracts of Phyllanthus niruri Linn. Plus Sida cordifolia Linn. In patients of diabetic sensory polyneuropathy. J Ayurveda Integr Med.

[55] Romero-Cerecero O., Zamilpa A., Tortoriello J. (2015). Effectiveness and tolerability of a standardized extract from Ageratina pichinchensis in patients with diabetic foot ulcer: a randomized, controlled pilot study. Planta Med.

[56] Sanpinit S., Chokpaisarn J., Na-Phatthalung P., Sotthibandhu D.S., Yincharoen K., Wetchakul P., Limsuwan S., Chusri S. (2024). Effectiveness of Ya-Samarn-Phlae in diabetic wound healing: evidence from in vitro studies and a multicenter randomized controlled clinical trial. J Ethnopharmacol.

[57] Soleimani Z., Hashemdokht F., Bahmani F., Taghizadeh M., Memarzadeh M.R., Asemi Z. (2017). Clinical and metabolic response to flaxseed oil omega-3 fatty acids supplementation in patients with diabetic foot ulcer: a randomized, double-blind, placebo-controlled trial. J Diabet Complicat.

[58] Tonaco L.A.B., Gomes F.L., Velasquez-Melendez G., Lopes M.T.P., Salas C.E. (2018). The proteolytic fraction from latex of Vasconcellea cundinamarcensis (P1G10) enhances wound healing of diabetic foot ulcers: a double-blind randomized pilot study. Adv Ther.

[59] Shataer D., Chen S., Wu Y., Liu F., Liu H., Lu J., Li B., Zhao L., Qiu S.X., Jumai A. (2025). Edible plant-derived xanthones as functional food components for metabolic syndrome mitigation: bioactivities and mechanisms. Foods.

[60] Qiu F., Fan S., Diao Y., Liu J., Li B., Li K., Zhang W. (2024). The mechanism of chebulae fructus immaturus promote diabetic wound healing based on network pharmacology and experimental verification. J Ethnopharmacol.

[61] Shataer D., Cao S., Liu X., Aierken K., Bhattacharya P., Sinha A., Liu H. (2025). Application of large language models in traditional Chinese medicine: a state-of-the-art review. Am J Chin Med.

