# Supplementary material for: Natural compounds for diabetes-related wounds or ulcers therapy: Evidence from preclinical and clinical studies
Source: J Ginseng Res. 2026 Feb 10;50(3):100990. doi: 10.1016/j.jgr.2026.100990 (PMC13149912; doi:10.1016/j.jgr.2026.100990)
Supplement: Multimedia component 1 [file mmc1.docx]

**Supplementary Table 1 Search strategy.**

| **The search strategy for PubMed** | |
| --- | --- |
| **Number** | **Search terms** |
| Clinical Trial | ((natural compound) OR (plant) OR (herb) OR (herbal product) OR (plant extract) OR (essential oil) OR (herbal constituent)) AND ((diabetes wound[Title/Abstract]) OR (diabetes ulcer[Title/Abstract]) OR (diabetic foot[Title/Abstract]) OR (diabetic wound healing[Title/Abstract])) AND (Clinical Study[Title/Abstract] OR Clinical Trial[Title/Abstract] OR Controlled Clinical Trial[Title/Abstract] OR Randomized Controlled Trial[Title/Abstract] OR Randomized[Title/Abstract] OR Controlled[Title/Abstract] OR Trial[Title/Abstract] OR Effect[Title/Abstract]) NOT ((research progress[Title/Abstract]) OR (review[Title/Abstract]) OR (meta[Title/Abstract]) OR (meta-analysis[Title/Abstract]) OR (systematic evaluation[Title/Abstract]) OR (rat[Title/Abstract]) OR (mouse[Title/Abstract]) OR (mice[Title/Abstract]) OR (animal[Title/Abstract])) |
| Non-Clinical Trial | ((natural compound) OR (plant) OR (herb) OR (herbal product) OR (plant extract) OR (essential oil) OR (herbal constituent)) AND ((diabetes wound[Title/Abstract]) OR (diabetes ulcer[Title/Abstract]) OR (diabetic foot[Title/Abstract]) OR (diabetic wound healing[Title/Abstract])) NOT (Clinical Study[Title/Abstract] OR Clinical Trial[Title/Abstract] OR Controlled Clinical Trial[Title/Abstract] OR Randomized Controlled Trial[Title/Abstract] OR Randomized[Title/Abstract] OR Controlled[Title/Abstract] OR Trial[Title/Abstract] OR Effect[Title/Abstract]) NOT ((research progress[Title/Abstract]) OR (review[Title/Abstract]) OR (meta[Title/Abstract]) OR (meta-analysis[Title/Abstract]) OR (systematic evaluation[Title/Abstract])) AND ((rat[Title/Abstract]) OR (mouse[Title/Abstract]) OR (mice[Title/Abstract]) OR (animal[Title/Abstract])) |
